# Evaluating the Polyphenol Profile in Three Segregating Grape (*Vitis vinifera* L.) Populations

**DOI:** 10.1155/2013/572896

**Published:** 2013-08-06

**Authors:** Alberto Hernández-Jiménez, Rocío Gil-Muñoz, Yolanda Ruiz-García, Jose María López-Roca, Adrián Martinez-Cutillas, Encarna Gómez-Plaza

**Affiliations:** ^1^Food Science and Technology Department, Faculty of Veterinary Science, University of Murcia, Campus de Espinardo, 30071 Murcia, Spain; ^2^Instituto Murciano de Investigación y Desarrollo Agroalimentario, Carretera La Alberca s/n, 30150 Murcia, Spain

## Abstract

This paper explores the characteristics of the anthocyanin and flavonol composition and content in grapes from plants resulting from intraspecific crosses of *Vitis vinifera* varieties Monastrell × Cabernet Sauvignon, Monastrell × Syrah, and Monastrell × Barbera, in order to acquire information for future breeding programs. The anthocyanin and flavonol compositions of twenty-seven hybrids bearing red grapes and 15 hybrids bearing white grapes from Monastrell × Syrah, 32 red and 6 white from Monastrell × Cabernet Sauvignon, and 13 red from Monastrell × Barbera have been studied. Among the intraspecific crosses, plants with grapes presenting very high concentrations of anthocyanins and flavonols were found, indicating a transgressive segregation for this character, and this could lead to highly colored wines with an increased benefits for human health. As regards the qualitative composition of anthocyanins and flavonols, the hydroxylation pattern of the hybrids that also may influence wine color hue and stability presented intermediate values to those of the parentals, indicating that values higher than that showed by the best parental in this respect will be difficult to obtain. The results presented here can be helpful to acquire information for future breeding efforts, aimed at improving fruit quality through the effects of flavonoids.

## 1. Introduction

**Figure 1:**
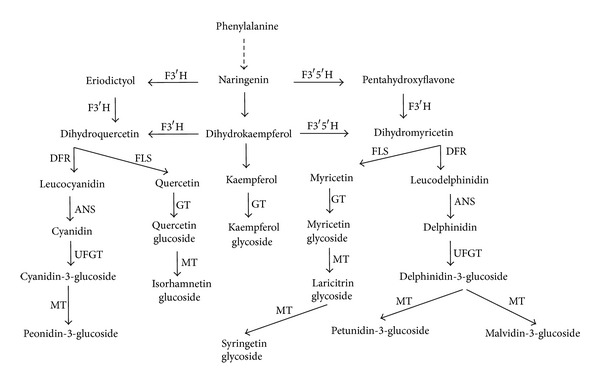
Flavonoid biosynthetic pathway. PAL: phenyl ammonia lyase, F3′H: flavonoid-3′-hydroxylase, F3′5′H: flavonoid-3′,5′-hydroxylase, UFGT: UDP-glucose: flavonoid 3-O-glucosyltransferase, MT: methyl transferase, DFR: dihydroxyflavanol-4-reductase, ANS: anthocyanin synthase, and FLS: flavonol synthase.

Anthocyanins are responsible for the color of red grape varieties and the wines produced from them. Flavonols are also important because they participate both in stabilizing anthocyanins in young red wines through copigmentation and in increasing the health-related properties of wine [1, 2]. Grape anthocyanins and flavonols are final products arising from the flavonoid biosynthetic pathway (Figure 1). *Vitis vinifera* varieties are characterized by the presence of 3-O-glucosides of delphinidin, peonidin, petunidin, cyanidin, and malvidin, together with their acylated derivatives [3]. The 3-O-glucosides of kaempferol, quercetin, and myricetin are the major flavonols in grapes as first reported by Cheynier and Rigaud [4] and recently confirmed by Castillo-Muñoz et al. [5], with quercetin glycosides usually being dominant [5], although a high presence of quercetin-3-O-glucuronide has also been observed in some varieties such as Petit Verdot [5, 6].

When studying grapes for winemaking, it is not only the quantity of anthocyanins that is important. The hydroxylation pattern of the B-ring of anthocyanins is one of the main structural features of flavonoids and is an important determinant of their coloration, stability, and antioxidant capacity. Trihydroxylated anthocyanins (delphinidin, petunidin, and malvidin-3-glucosides) are more stable in wines than dihydroxylated ones (cyanidin and peonidin-3-glucosides) [7]. Those with orthodiphenolic groups (cyanidin, delphinidin, and petunidin) have an enhanced susceptibility to oxidation. The methoxylated anthocyanins are also more stable. The same applies to acylated anthocyanins, since their esterification of anthocyanins promotes intramolecular aggregation or stacking, which protects the oxonium ion from decomposition [8]. 

As hydroxylation of the B-ring is carried out by flavonoid-3′-hydroxylase (F3′H) and flavonoid-3′,5′-hydroxylase (F3′5′H) enzymes, the composition of anthocyanins in grape skins will be determined by the relative activities of these enzymes. At the same time, methyl transferase (MT) activity will determine the different methoxylation patterns of B ring and acyl transferase the presence of acyl derivatives. Most of these enzymes also control the synthesis of flavonols. 

Plant adaptation to different environments and centuries of selection by humans has produced numerous genotypes in which the intensity of the red coloration varies extensively. A mixture of variation in anthocyanin content and the relative proportion of different anthocyanins can produce different phenotypes for skin pigmentation with consequent technological and nutritional differences [9]. Also, these differences could be a very useful chemotaxonomical tool [3]. 

At present, cross-breeding and bud mutation are still the most common way for developing new wine grape cultivars [10]. The determination of the mechanisms of the inheritance of the flavonoid composition could help in the election of the parentals in the breeding programs. The objective of this study therefore was to explore the characteristics of the anthocyanin and flavonol composition and content in intraspecific hybrids of Monastrell × Syrah, Monastrell × Cabernet Sauvignon, and Monastrell × Barbera, in order to acquire information for future breeding efforts aimed at improving fruit quality through the effects of flavonoids and to provide an insight into the mechanisms that control the inheritance of flavonoid characteristics among hybrids. 

## 2. Material and Methods

A collection of plants arising from crosses from Monastrell × Syrah, Monastrell × Cabernet Sauvignon, and Monastrell × Barbera was used in this study. The study was conducted in an experimental vineyard of 1 ha located in Bullas (Murcia, SE Spain). The parentals (Monastrell, Syrah, Cabernet Sauvignon, and Barbera) were planted in 1997, whereas the seeds for the interspecific hybrids were planted in 2000. The training system was a bilateral cordon trellised to a three-wire vertical system, and drip irrigation was applied. Planting density was 2.5 m between rows and 1.25 m between vines. Two two-bud spurs (4 nodes) were left at pruning time. Grapes were sampled in 2007. 

The plants derived from Monastrell self-pollination and from pollen donors other than Syrah, Cabernet Sauvignon, or Barbera were identified genetically and discarded using the microsatellite (SSR, Simple Sequence Repeat) loci segregating 1 : 1 : 1 : 1 according to Bayo-Canha et al. [11]. Total DNA was extracted from approximately 20 mg of young frozen leaves using a DNeasy Plant Mini Kit (Qiagen, Valencia, CA, USA) following the manufacturer's protocol. Genotyping was carried out as described in Adam-Blondon et al. [12]. PCR products were separated by capillary electrophoresis performed on an ABI Prism 3100 genetic analyzer (Applied Biosystems, Carlsbad, CA, USA), and the fragments were sized using GeneMapper software (Applied Biosystems, Carlsbad, CA, USA). 

Grapes were harvested at a total of soluble solids content between 23 and 27°Brix. The sampling was randomly made by picking berries from the top, central, and bottom parts of several clusters of each hybrid vine. The size of the sample was around 300 berries, which were bulked and separated in 3 subsamples of approximately 100 berries to run triplicate analyses. Grape samples were kept frozen (−20°C) until extraction and analysis.

### 2.1. Anthocyanin Monoglycosides and Flavonols in Berry Skins

Grapes were peeled with the help of a scalpel. Samples (2 g) were immersed in methanol (40 mL) in hermetically closed tubes and placed on a stirring plate at 150 rpm and 25°C. After 2 hours, the methanolic extracts were acidified with 5% formic acid (1 : 2 v/v), filtered through 0.2 *μ*m PTFE filters, and analysed by HPLC.

### 2.2. Identification and Quantification of Anthocyanins

The HPLC analyses were performed on a Waters 2695 liquid chromatograph (Waters, PA, USA), equipped with a Waters 2996 diode array detector and a Primesep B2 column (Sielc, Illinois, USA), 25 × 0.4 cm, 5 *μ*m particle size, using as solvents water plus 5% formic acid (solvent A) and HPLC grade acetonitrile (solvent B) at a flow rate of 0.8 mL min^−1^. Elution was performed with a gradient starting with 5% B to reach 9% B at 28 min, 13% B at 30 min, 21% B at 52 min, 24% B at 65 min, and 70% B at 75 min, maintaining this gradient for 5 minutes. Chromatograms were recorded at 520 nm (anthocyanins) and 360 nm (flavonols). 

Identification of the compounds was carried out by comparing their UV spectra recorded with the diode array detector and those reported in the literature. Also, an HPLC-MS analysis was made to confirm the identity of each peak. An LC-MSD-Trap VL-01036 liquid chromatograph-ion trap mass detector (Agilent Technologies, Waldbronn, Germany) equipped with an electrospray ionization (ESI) system was used. Elution was performed in the HPLC analysis conditions described previously, with a flow rate of 0.8 mL min^−1^. The heated capillary and voltage were maintained at 350°C and 4 kV, respectively. Mass scans (MS) were measured from *m*/*z* 100 up to *m*/*z* 800. 

 Anthocyanins were quantified at 520 nm as malvidin-3-glucoside, using malvidin-3-glucoside chloride as external standard (Extrasynthèse, Genay, France). Flavonols were quantified at 360 nm as quercetin-3-glucoside, using this compound as external standard (Sigma, Missouri, USA).

### 2.3. Statistical Data Treatment

All the analyses were performed with the statistical package Statgraphics 5.1.

## 3. Results and Discussion

For this study, 27 hybrids bearing red grapes and 15 hybrids bearing white grapes from Monastrell × Syrah, 32 red and 6 white from Monastrell × Cabernet Sauvignon, and 13 red from Monastrell × Barbera were studied. The presence of white hybrids in Monastrell × Cabernet Sauvignon and Monastrell × Syrah indicates the heterozygous nature of the parentals in regard to genes controlling anthocyanin synthesis, whereas Barbera, being homozygous [13], does not produce hybrids bearing white grapes. Boss et al. [14] and Kobayashi et al. [15] showed that expression of the UDP-glucose: flavonoid 3-O-glucosyltransferase (UFGT) gene is critical for anthocyanin biosynthesis in grape. Experiments with the berry skins of white and red cultivars revealed that the UFGT gene was expressed in all the red cultivars but not in the white ones whereas the other genes involved in anthocyanin biosynthesis (Figure 1) are expressed in both white and red cultivars. The presence or absence of the enzyme UFGT is controlled by Myb-related regulatory genes, and the insertion of the retroelement *Gret1* in the promoter region of *VvmybA1* gene appears to be associated with white-fruited cultivars when present in a homozygous state. Pigmented cultivars possess at least one allele at the *VvmybA1* locus not containing this large retroelement [13, 16], as is the case of parentals of this study.

**Figure 2:**
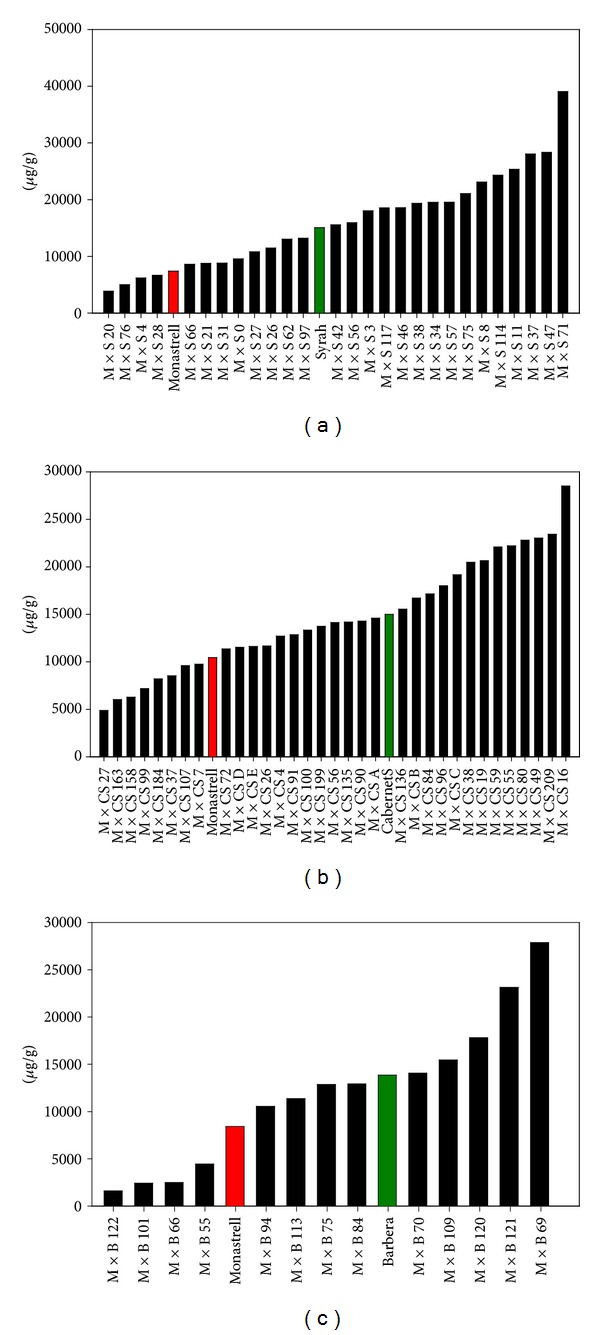
Concentration of anthocyanins in Monastrell × Syrah (a), Monastrell × Cabernet Sauvignon (b), and Monastrell × Barbera (c) hybrid grapes (each bar represents the mean value of three samples).

Table 1 shows the results of the anthocyanin analysis for the studied grapes and Figure 2 the range of concentrations among the hybrids. Syrah grapes contained higher concentration of anthocyanins than Monastrell grapes. The mean concentration in their hybrid grapes (16.31 mg/g fresh skin) was slightly higher than in Syrah (15.06 mg/g fresh skin), and the maximum value reached by the grapes of one seedling (39.49 mg/g fresh skin) was twice the value found in Syrah. Cabernet Sauvignon and Barbera grapes also showed higher concentration, of anthocyanins than Monastrell and were similar to that of Syrah grapes. The mean values of anthocyanin content in the grapes of their seedlings were slightly lower than in Cabernet Sauvignon and Barbera (14.42 and 12.08 mg/g fresh skin respectively) and higher than that shown by Monastrell grapes (7.40 mg/g fresh skin), and, again, the maximum value found in their seedlings was double that of Cabernet Sauvignon and Barbera grapes. Many hybrids presented grapes with much higher concentration than their parentals as can be seen in Figure 2. The appearance of a large number of hybrids in which the anthocyanin concentration is not within the range of concentration of their parental phenotypes is called transgressive segregation, frequent in intraspecific crosses and in domesticated populations. The occurrence of the segregation of a given trait manifested mainly in one direction (as happens in our case, most of the hybrids showing higher values of anthocyanin concentration than the parentals) may imply that the trait has undergone fairly constant directional selection or a certain overdominance of the genes controlling phenolic synthesis [17, 18]. Similar results were found by Liang et al. [10] in grapes, but our findings differ from those of Ju et al. [19] in apples that found that crossing between two red-fruited apple cultivars produced less colored progeny. A previous work exploring the proanthocyanidin content of Monastrell × Syrah hybrid grapes also reported this type of segregation for this character [20].

**Figure 3:**
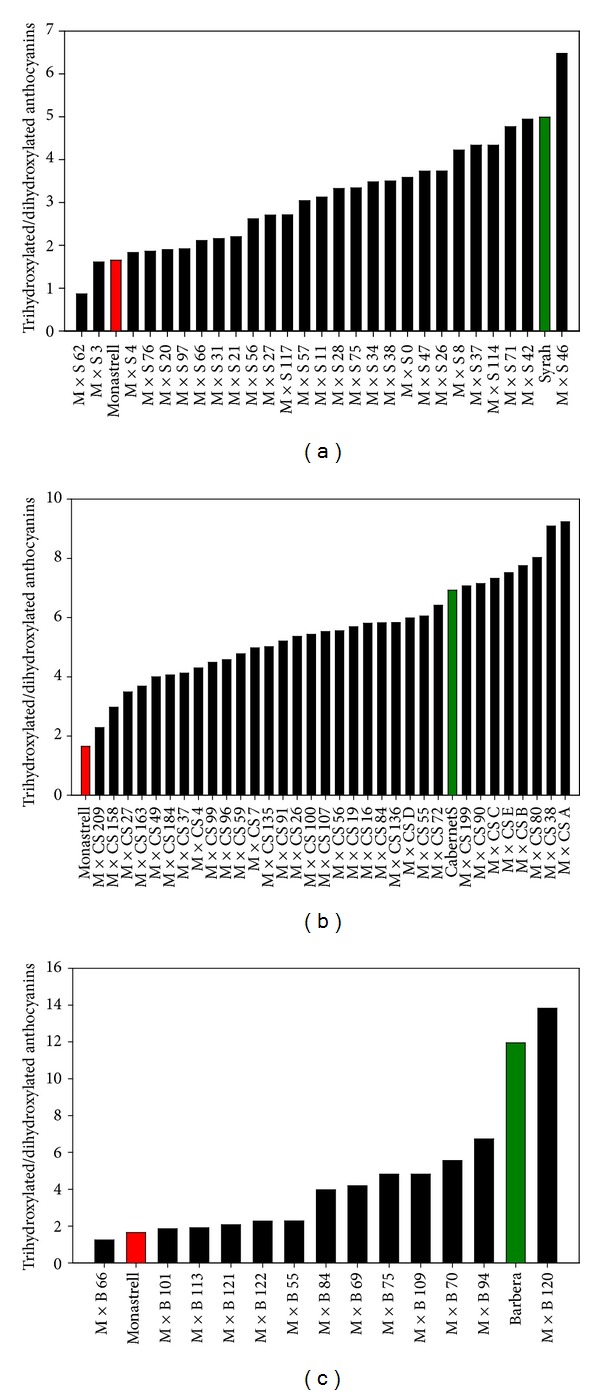
Trihydroxylated/dihydroxylated anthocyanins ratios in Monastrell × Syrah (a), Monastrell × Cabernet Sauvignon (b), and Monastrell × Barbera (c) hybrid grapes.

As stated previously, the presence of the enzyme UFGT is necessary for anthocyanin biosynthesis. However, the biosynthesis of the different anthocyanin precursors is driven upstream of the enzyme UFGT by the activity of F3′H and F3′5′H enzymes, which add either a single hydroxyl group or two to dihydrokaempferol (Figure 1). Once converted to dihydroquercetin or dihydromyricetin, these intermediates flow through common downstream enzymes to form disubstituted and trisubstituted anthocyanins, when UFGT is expressed, and to form other polyphenols (flavanols, flavonols) at different developmental stages. All the studied hybrids bearing red grapes synthesised all five anthocyanins (the dihydroxylated cyaniding, peonidin-3-glucosides, the trihydroxylated delphinidin, petunidin, and malvidin-3-glucosides), together with their acylated derivatives. This means that all the parentals and the hybrids expressed functional F3′H and F3′5′H genes for the synthesis of 3′4′-OH and 3′4′5′-OH anthocyanins, as well as methyltransferases (*MT*) for the methylation of primary anthocyanins. As regards the percentage of the different anthocyanins in the different parentals, Monastrell grapes were characterized by a high percentage of cyanidin, suggesting a lower F3′5′H activity than in the other varieties. A low expression of F3′5′H has been associated with cyanidin-based anthocyanins in grape leafs [21]. The percentages of malvidin-based anthocyanins in Monastrell did not exceed 40%, so the total percentage of trihydroxylated anthocyanins was low. The percentage of trihydroxylated anthocyanins reached 83% in Syrah grapes, 87.4% in Cabernet Sauvignon grapes, and 92.3% in Barbera grapes. The percentage of trihydroxylated anthocyanins was even higher in some Monastrell × Cabernet Sauvignon and Monastrell × Barbera hybrids, reaching values as high as 90.5% and 93%, respectively. The mean value of the percentage of trihydroxylated anthocyanins in the hybrid grapes is close to the mean value between both parentals. The segregation can be fitted to a normal distribution, and a very low number of hybrids presented higher or lower values than in the parentals, as can be seen in Figure 3. These results were similar to those obtained during the first screening of the anthocyanin profile in Monastrell × Cabernet Sauvignon grapes [22]. In spite of the presence of all anthocyanin biosynthetic enzymes in all the investigated hybrids (since all the possible structures were found), a genotype-specific regulation of the structural genes along the core pathway and at the main branching points is presumed to underlie the observed methoxylation and hydroxylation variations among the grapes from the parentals and those of their hybrid plants.

With regard to the percentage of anthocyanin acylation, Barbera grapes showed the lowest percentage of acylation (34.3%), followed by Monastrell (44.8%), and Cabernet Sauvignon showed the highest percentages (54%). The mean values of the percentage of acylated anthocyanins for Monastrell × Cabernet Sauvignon hybrids were 47%, 56.8% for Monastrell × Syrah (higher than the percentage found in Syrah) while the hybrid grapes from Monastrell × Barbera showed the lowest percentages of acylation (28%). As in the case of the anthocyanin concentration data, there was a tendency towards higher values of acylation in the hybrids, and no hybrid contained only nonacylated anthocyanins. 

As regards flavonols (Table 2), these flavonoids were present in both white and red grapes. We could identify mono- (kaempferol), di- (quercetin and isorhamnetin), and trihydroxylated (myricetin, laricitrin, and syringetin) flavonol glycosides (glucosides, glucoronides, and small quantities of galactosides). In red grapes, the monohydroxylated flavonols represented the lowest percentage, especially in Barbera grapes (4.3%). Monastrell grapes presented a very high percentage of dihydroxylated flavonols (70.7%), much higher than in the other varieties and, therefore, a lower percentage of trihydroxylated flavonols whereas Barbera, Syrah, and Cabernet Sauvignon grapes reached a percentage of trihydroxylated flavonols of around 45–50%. This fact indicates lower F3′5′H activity in Monastrell grapes, as also observed for the anthocyanins. 

**Figure 4:**
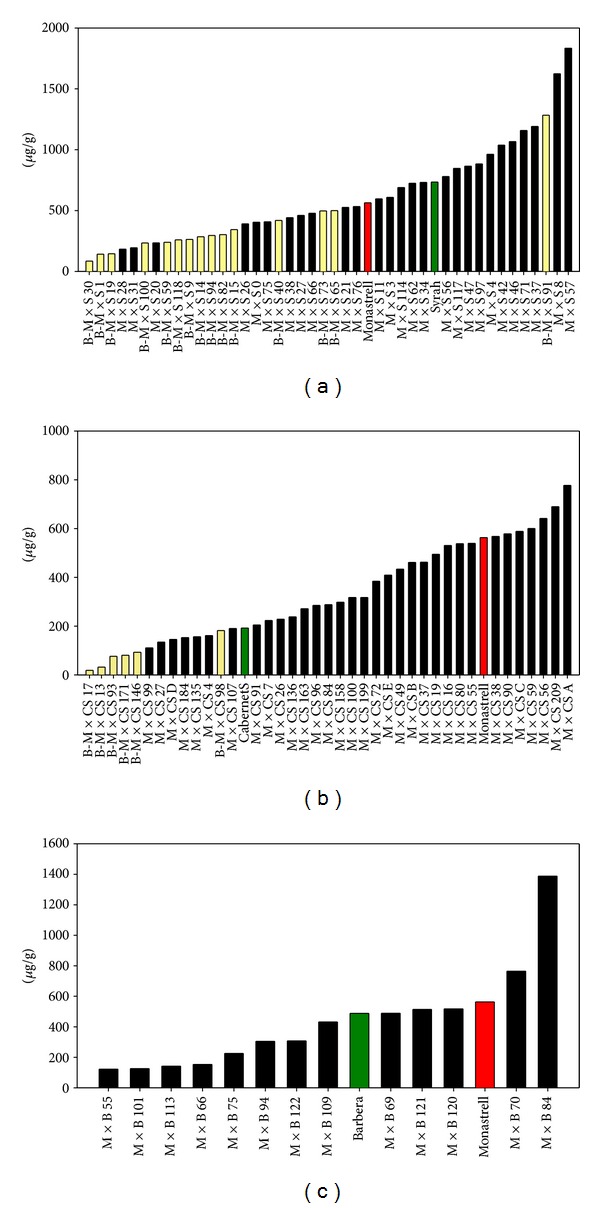
Concentration of flavonols in Monastrell × Syrah (a), Monastrell × Cabernet Sauvignon (b), and Monastrell × Barbera (c) hybrid grapes (yellow bars indicate white grape bearing hybrids).

As regards the flavonol content, Cabernet Sauvignon grapes showed low concentrations of flavonols and Monastrell and Syrah much higher concentrations while the highest value was found in red grapes from a hybrid plant from Monastrell × Syrah (Table 2, Figure 4), reaching values as high as 1.83 mg/g of skin. In the hybrids bearing white grapes, a lower concentration of flavonols was measured compared with that of the hybrids bearing red grapes. Azuma et al. [23, 24] stated that Myb genes, besides the regulation of the UFGT expression, appear to enhance the expression of all the genes involved in the anthocyanin biosynthesis pathway because the transcription of all anthocyanin biosynthesis genes appears to be slightly activated, which would explain the higher concentration of flavonols in red grapes. Also, in these white skinned grapes, trihydroxylated flavonols were barely present. Mattivi et al. [25], studying the flavonol profile of several grape varieties, did not detect trihydroxylated flavonols in white grapes. Bogs et al. [26] did not find significant expression of F3′5′H and UFGT in white grape varieties, which suggests a related regulation of F3′5′H and UFGT during berry ripening and justifies the almost null presence of trihydroxylated flavonols in white grapes. F3′5′H was detected in white grapes var. Chardonnay but prior to veraison [26] since it is needed for flavanol biosynthesis, which seems to be controlled differently; indeed, no differences in the percentage of trihydroxylated flavanols were observed between the red and white grapes arising from the cross of Monastrell × Syrah [20]. However, other studies stated that flavonol synthase (FLS) was not upregulated when UFGT was expressed and that the increase in flavonols in red grapes was a consequence of an increase flux through the flavonoid pathway [27]. 

## 4. Conclusions

The study of the anthocyanin and flavonol profiles of the grapes from the hybrid plants can be useful for a targeted informative metabolomic analysis [28], a tool for selecting promising grapes according to their profile and/or content.

Seedlings with grapes presenting very high concentrations of anthocyanins and flavonols can be expected from intraspecific crosses, and these resulting grapes could lead to highly colored wines, with increased health-related properties. In this way, three plants arising from Monastrell × Syrah (hybrids 8, 37, and 71) presented anthocyanin and flavonol concentrations higher than 20 and 1 mg/g fresh skin, respectively (Figures 2 and 4). Also, four plants arising from Monastrell × Cabernet Sauvignon (hybrids 38, 59, 55, and 80) and two from Monastrell × Barbera (hybrid plants 120, 121) showed anthocyanin values higher than 20 mg/g fresh skin accompanied with high concentration of flavonols (Figures 2 and 4). The hydroxylation pattern, which also influences wine color and its stability, will be strongly influenced by the parentals pattern since values higher than that shown by the best parental in this respect will be difficult to obtain. Also, in the case of the crosses between heterozygous parentals (Monastrell, Cabernet Sauvignon, and Syrah) hybrids bearing white grapes can be obtained, some of them with a high concentration of flavonols, that could be of importance in the health properties of this fruit and its derived products, such as the wine. The information obtained in this study should be helpful for selecting parentals for breeding programs.

## Figures and Tables

**Table 1:** Mean values (*n* = 3) of the anthocyanin profile and total anthocyanin content (mg/g fresh skin) in Monastrell, Syrah and Cabernet, Sauvignon grapes and their hybrids.

	% del.	% cyan.	% pet.	% peon.	% malv.	% nonacylated	% acylated	% dihydrox.	% trihydrox.	Total (mg/g)
Monastrell										
Mean	12.5	13.7	10.0	20.0	39.7	55.2	44.8	37.7	62.3	7.40
Syrah										
Mean	7.2	3.6	8.1	10.9	68.1	49.6	50.3	16.7	83.3	15.06
CS										
Mean	9.6	5.2	10.1	7.4	67.7	45.9	54.1	12.6	87.4	15.07
Barbera										
Mean	9.8	3.1	13.9	4.7	68.6	65.6	34.3	7.7	92.3	14.02
Mon. × Sy. (27)^a^										
Mean	10.0	7.1	8.6	15.4	54.8	43.2	56.8	26.5	73.4	16.31
Minimum	5.7	3.2	6.4	6.5	29.5	46.2	29.7	13.2	46.2	3.86
Maximum	24.3	15.4	13.9	37.5	72.7	70.2	71.3	53.7	86.8	39.49
Mon. × CS. (32)^b^										
Mean	12.4	6.0	13.3	10.5	57.8	52.5	47.2	16.5	83.5	14.44
Minimum	7.5	3.5	9.4	5.6	44.4	35.2	31.3	9.5	68.9	4.87
Maximum	23.4	12.1	20.3	21.5	68.5	68.6	64.7	31.1	90.5	28.67
Mon. × Bar. (13)^c^										
Mean	10.9	7.7	13.3	16.2	51.9	72.0	28.0	23.9	76.1	12.08
Minimum	4.1	1.1	9.0	4.4	31.7	41.5	10.1	6.7	55.2	1.61
Maximum	17.0	14.6	19.7	36.4	77.9	89.8	58.5	44.7	93.3	27.88

^a,b,c^The number in parenthesis represents the number of hybrids for each crossing.

% del.: percentage of delphinidin derivatives, % cyan.: percentage of cyanidin derivatives, % pet.: percentage of petunidin derivatives, % peon.: percentage of peonidin derivatives, % malv.: percentage of malvidin derivatives, % nonacylated: percentage of nonacylated anthocyanins, % acylated.: percentage of acylated anthocyanins, % dihydrox.: percentage of dihydroxylated anthocyanins, % trihydrox.: percentage of trihydroxylated anthocyanins, and Total (mg/g): total anthocyanin content (mg per g of skin).

**Table 2:** Mean values of the flavonol profile and total flavonol content (mg/g fresh skin) in Monastrell, Syrah, and Cabernet Sauvignon grapes and their hybrids (*n* = 3).

	% monohydr.	% dihydr.	% trihydrox.	Total
Monastrell				
Mean	10.9	70.7	18.4	0.56
Syrah				
Mean	13.4	44.2	42.4	0.73
CS.				
Mean	14.7	44.6	40.7	0.19
Barbera				
Mean	4.3	53.3	42.4	0.49
Red hybrids				
Mon. × Sy.				
Mean	10.4	52.4	37.2	0.73
Minimum	5.3	36.3	16.5	0.18
Maximum	16.0	72.6	55.0	1.83
Mon. × CS.				
Mean	11.3	51.1	37.6	0.37
Minimum	5.0	35.2	20.3	0.11
Maximum	20.9	65.0	58.3	0.78
Mon. × Bar.				
Mean	9.4	61.9	28.7	0.42
Minimum	4.7	42.9	8.7	0.12
Maximum	13.6	83.9	43.4	1.39
White hybrids				
Mon. × Sy.				
Mean	13.4	84.1	2.4	0.35
Minimum	4.1	74.6	0.6	0.08
Maximum	23.7	94.7	7.2	1.28
Mon. × CS.				
Mean	20.0	74.8	5.1	0.08
Minimum	16.8	68.9	0.9	0.02
Maximum	25.9	78.4	12.7	0.18

% monohydrox.: percentage of monohydroxylated flavonols, % dihydrox: percentage of dihydroxylated flavonols, % trihydrox.: percentage of trihydroxylated flavonols, Total: total flavonol content (mg per g of skin).
